# Delineating the effects of 5-fluorouracil and follicle-stimulating hormone on mouse bone marrow stem/progenitor cells

**DOI:** 10.1186/s13287-016-0311-6

**Published:** 2016-04-19

**Authors:** Ambreen Shaikh, Deepa Bhartiya, Sona Kapoor, Harshada Nimkar

**Affiliations:** Stem Cell Biology Department, National Institute for Research in Reproductive Health (ICMR), Jehangir Merwanji Street, Parel, Mumbai, 400 012 India

**Keywords:** Very small embryonic-like stem cells, Hematopoietic stem cells, Follicle-stimulating hormone, 5-Fluorouracil, OCT-4, Bone marrow

## Abstract

**Background:**

Pluripotent, Lin^–^/CD45^–^/Sca-1^+^ very small embryonic-like stem cells (VSELs) in mouse bone marrow (BM) are resistant to total body radiation because of their quiescent nature, whereas Lin^–^/CD45^+^/Sca-1^+^ hematopoietic stem cells (HSCs) get eliminated. In the present study, we provide further evidence for the existence of VSELs in mouse BM and have also examined the effects of a chemotherapeutic agent (5-fluorouracil (5-FU)) and gonadotropin hormone (follicle-stimulating hormone (FSH)) on BM stem/progenitor cells.

**Methods:**

VSELs and HSCs were characterized in intact BM. Swiss mice were injected with 5-FU (150 mg/kg) and sacrificed on 2, 4, and 10 days (D2, D4, and D10) post treatment to examine changes in BM histology and effects on VSELs and HSCs by a multiparametric approach. The effect of FSH (5 IU) administered 48 h after 5-FU treatment was also studied. Bromodeoxyuridine (BrdU) incorporation, cell cycle analysis, and colony-forming unit (CFU) assay were carried out to understand the functional potential of stem/progenitor cells towards regeneration of chemoablated marrow.

**Results:**

Nuclear OCT-4, SCA-1, and SSEA-1 coexpressing LIN^–^/CD45^–^ VSELs and slightly larger LIN^–^/CD45^+^ HSCs expressing cytoplasmic OCT-4 were identified and comprised 0.022 ± 0.002 % and 0.081 ± 0.004 % respectively of the total cells in BM. 5-FU treatment resulted in depletion of cells with a 7-fold reduction by D4 and normal hematopoiesis was re-established by D10. Nuclear OCT-4 and PCNA-positive VSELs were detected in chemoablated bone sections near the endosteal region. VSELs remained unaffected by 5-FU on D2 and increased on D4, whereas HSCs showed a marked reduction in numbers on D2 and later increased along with the corresponding increase in BrdU uptake and upregulation of specific transcripts (Oct-4A, Oct-4, Sca-1, Nanog, Stella, Fragilis, Pcna). Cells that survived 5-FU formed colonies in vitro. Both VSELs and HSCs expressed FSH receptors and FSH treatment enhanced hematopoietic recovery by 72 h.

**Conclusion:**

Both VSELs and HSCs were activated in response to the stress created by 5-FU and FSH enhanced hematopoietic recovery by at least 72 h in 5-FU-treated mice. VSELs are the most primitive pluripotent stem cells in BM that self-renew and give rise to HSCs under stress, and HSCs further divide rapidly and differentiate to maintain homeostasis. The study provides a novel insight into basic hematopoiesis and has clinical relevance.

**Electronic supplementary material:**

The online version of this article (doi:10.1186/s13287-016-0311-6) contains supplementary material, which is available to authorized users.

## Background

In the last decade various groups have reported the presence of non-hematopoietic stem cells in the bone marrow (BM) [1–5] using an array of cell surface markers. In 2006, a rare population of these cells expressing pluripotent markers Oct-4 and Nanog was identified and well characterized in the bone marrow [6]. Over the decade these LIN^–^/CD45^–^/SCA-1^+^ in mice and CD133^+^ in humans, very small embryonic-like stem cells (VSELs) have been described in various murine and human adult tissues including bone marrow [7, 8], cord blood [9–11], testis [12, 13], ovary [14, 15], uterus [16], pancreas [17], and other organs [18, 19]. True to their pluripotent nature, the human and murine VSELs under specific conditions give rise to cells of all the three germ layers in vitro [6, 20, 21]. VSELs are quiescent in nature owing to partial erasure of imprinted genes [22, 23] and thus do not either expand easily in vitro or form teratoma. However, several reports have shown that VSELs enter the cell cycle and mobilize in response to stress or tissue injury [24–30]. In the hematopoietic system, VSELs are considered to be at the top of the hierarchy, because the CD45^–^ VSELs in vitro give rise to the CD45^+^ hematopoietic stem cells (HSCs) [31, 32]. Till date, however, the role of VSELs in hematopoiesis in vivo either in steady-state conditions or in response to bone marrow injury has not been studied extensively.

Ratajczak’s group [6] was the first to study VSELs (LIN^–^/CD45^–^/SCA-1^+^) and HSCs (LIN^–^/CD45^+^/Sca-1^+^) in mouse BM by flow cytometry. The main distinction between the two cell types is the presence or absence of CD45 expression. CD45 is pan-hematopoietic marker which is expressed by all leukocytes, including HSCs [33]. Others have also reported that cells which express CD45 and CD34 but lack CD38 and lineage antigens (CD45^+^CD34^+^CD38^–^Lin^–^) are HSC populations [34] and LIN^–^/CD45^–^/SCA-1^+^ are VSELs [20]. Being pluripotent and not committed to the hematopoietic fate, VSELs do not express CD45, are negative for LIN markers, and rather express pluripotent markers. Besides the cell surface marker profile, VSELs and HSCs are also distinguished from each other based on their size and OCT-4 expression pattern. Oct-4 is a nuclear transcription factor crucial to maintain the pluripotent state and its gene is known to be alternatively spliced and has pseudogenes [35–38]. OCT-4 isoform 1 (transcript Oct-4A) is localized in the nucleus, maintains stemness properties, and confers self-renewal function, whereas OCT-4 isoform 2 (transcript Oct-4B) is localized in the cytoplasm and has no assigned biological function as yet [39]. We have reported VSELs with nuclear OCT-4A and slightly bigger progenitors with cytoplasmic OCT-4 in adult testis (spermatogonial stem cells (SSC)) [12], ovary (ovarian stem cells (OSC)) [14], and Wharton’s jelly (mesenchymal cells (MSC)) [40] using a polyclonal OCT-4 antibody which detects both of the isoforms. Based on the OCT-4 staining pattern, we have postulated that immediate descendants or “progenitors” (SSCs, OSCs, MSCs) with cytoplasmic OCT-4 supposedly arise from the pluripotent VSELs. Nuclear form of OCT-4A is no longer required when VSELs initiate differentiation, in the progenitors it is expressed in the cytoplasm, and eventually get degraded as cells differentiate further. Besides OCT-4, VSELs also express several other pluripotent and primordial germ cell markers and thus can easily be distinguished from the HSCs.

Pietras et al. described the heterogeneity that exists among HSCs related to the degree of quiescence. At any given moment, 90–95 % of HSCs exhibit an “activated” phenotype and only 5–10 % are “dormant” [41]. In contrast, all VSELs are quiescent in nature and as a result survive the effects of irradiation and chemotherapy. Ratajczak et al. [31] reported that 1000–1500 cGY total body irradiation in mice resulted in the loss of HSCs whereas VSELs survived and 12 % of them incorporated bromodeoxyuridine (BrdU). Similarly, VSELs survived and their percentage increased in mouse ovaries [15] and testis [13] on treatment with busulphan and cyclophosphamide. Recently we have shown that cord-blood VSELs, which are otherwise quiescent, also enter the cell cycle in response to 24 h of 5-fluorouracil (5-FU) treatment in vitro [11]. The aim of the present study was to understand the differential effects of 5-FU on bone marrow stem/progenitor cells. 5-FU (150 mg/kg) treatment depleted cycling cells, thereby creating stress in the mouse bone marrow and further experiments were undertaken to study whether recovery of bone marrow involved only HSCs or also the VSELs.

Recent reports have shown that, along with cytokines and growth factors, the long-range signaling hormones also affect HSC activity [42]. The hematopoietic progenitors respond in vitro by proliferation to estrogen and androgens [43–45]. Administration of estradiol and an increased level of estrogen as seen during pregnancy also affect survival, self-renewal, and differentiation of HSCs [45]. Accumulating evidence suggests that along with hematopoietic progenitors even VSELs in mouse BM and human cord blood express receptors for sex hormones [46, 47]. Mierzejewska et al. [46] reported that 10-day administration of hormones (follicle-stimulating hormone (FSH), luteinizing hormone, prolactin, androgen, and estrogen) stimulated proliferation of VSELs and HSPCs in vivo as evaluated by the uptake of BrdU. Another study has also recently reported effective mobilization of VSELs and HSCs into circulation in 15 women being treated with FSH to stimulate ovaries in an infertility clinic [48]. Our group had initially reported expression of FSH receptor (FSHR) on ovarian VSELs and OSCs and their modulation by FSH and recently observed similar FSHR expression on testicular VSELs and SSCs and their stimulation by FSH [15, 49–52].

In the present study, different approaches have been used to further characterize and describe the dynamics and functional potential in vitro of VSELs and HSCs in normal and 5-FU-treated adult mouse bone marrow. In addition, the influence of FSH on the recovery of BM after 5-FU treatment and whether FSH stimulates self-renewal, expansion, and/or differentiation of VSELs and HSCs were studied.

## Methods

All experimental protocols used in the present study were approved by the Institutional Animal Ethics Committee of NIRRH. Adult Swiss mice 6–8 weeks old maintained in the institute experimental animal facility were used for the study. They were housed in a temperature- and humidity-controlled room on a 12-h light/12-h darkness cycle with free access to food and water.

### Experimental design

#### Characterization of VSELs and HSCs in adult mouse BM

Total nucleated cells obtained from mouse BM were studied for VSELs and HSCs by various methods including flow cytometry (based on size and marker expression: VSELs are 3–5 μm and LIN^–^/CD45^–^/SCA-1^+^, whereas HSCs are ≥6 μm and LIN^–^/CD45^+^/SCA-1^+^), immunolocalization to study pluripotent (OCT-4, SSEA-1, SCA-1), primordial germ cell (STELLA), and proliferation (PCNA) specific markers, and quantitative RT-PCR studies for studying pluripotent (Oct-4A, Nanog, Tert), primordial germ cell (Stella, Fragilis), Sca-1, Oct-4, and proliferation (Pcna) specific transcripts. Due care was taken to design Oct-4 primers which were designed from exon 1 to specifically detect Oct-4A and spanning exons 2–4 to detect total Oct-4 (which majorly includes Oct-4B) as described earlier [15]. Complete lists of the antibodies and primers used in the study are provided in Additional file 1: Tables S1 and S2 respectively.

#### Effect of 5-FU treatment on mouse BM stem/progenitor cells

Mice were treated with 150 mg/kg body weight of 5-FU (Biochem, Mumbai, India) via the intraperitoneal route. The animals were sacrificed 2, 4, and 10 days (D2, D4, and D10) after the treatment by cervical dislocation and the harvested BM was processed for characterization studies as already described along with histological studies. Besides studying the effect on VSELs and HSCs, flow cytometry analysis was also performed to study the effect of 5-FU on proliferation of various subsets of cells by BrdU uptake and cell-cycle status. The cells that survived 5-FU treatment were also cultured in Methocult medium to study their functional potential.

#### Effect of FSH treatment on stem/progenitor cells in 5-FU-treated mice

To study the effect of FSH on BM cells in 5-FU-treated mice, recombinant FSH (5 IU, Gonal F, 10 U; Merck Serono, Switzerland) was injected subcutaneously 48 h after animals were treated with 5-FU. BM was harvested after 2 and 5 days of FSH treatment and processed for various studies as already described. Presence of FSH receptors on various cell types was also studied by flow cytometry and immunolocalization studies.

#### Methods

##### Isolation of total nucleated cells from mouse BM

BM cells from 6–8-week-old adult Swiss mice were isolated. Briefly, BM was flushed from tibias and femurs using DMEM-F12 media (Gibco, Carlsbad, CA, USA) and the cells were passed through 70 μm filter (Falcon, Corning, NY, USA). The filtrate was centrifuged at 1000 × *g* for 10 min and the pellet obtained was resuspended in 1× RBC lysis buffer (hypotonic ammonium chloride solution) for 10 min. A population of total nucleated cells (TNCs) was obtained after lysis of RBCs and washed twice with DMEM-F12 + 2 % fetal bovine serum (FBS; Gibco). TNCs obtained by this method were used for various studies.

##### Flow cytometry

BM cells from normal, 5-FU-treated, and 5-FU + FSH-treated mice were used for flow cytometry to enumerate Sca-1^+^/Lin^–^/CD45^–^ VSELs and Sca-1^+^/Lin^–^/CD45^+^ HSCs using the gating strategy described by Kucia et al. [6]. A single-cell suspension was prepared and stained with FITC-conjugated rat anti-mouse SCA-1 (BD Biosciences, San Jose, CA, USA), PE rat anti-mouse CD45 (BD Biosciences), and APC mouse Lineage antibody cocktail (BD Pharmingen, San Diego, CA, USA) for 60 min on ice. After washing, the stained cells were run on FACS Aria (BD Biosciences). At least 10^5^ events were acquired and results were analyzed by using BD FACS Diva software (BD Biosciences).

##### BrdU staining

Proliferation events in BM cell populations were examined by BrdU incorporation in normal and 5-FU-treated mice by flow cytometry. Briefly, after 5-FU and 5-FU + FSH treatment, the mice were injected with BrdU (1 mg, intraperitoneal; Sigma-Aldrich, St. Louis, MO, USA) daily and a final injection of BrdU was administered 1 h before sacrifice. BM was subsequently isolated and TNCs were immunostained for CD45, LIN markers, SCA-1, and BrdU (FITC BrdU Flow Kit; BD Pharmingen). The manufacturer’s protocol was followed and the stained cells were run on FACS Aria. The results obtained were analyzed using FACS Diva software.

Detailed descriptions of the other methods used are presented in Additional file 1.

##### Statistical analysis

Arithmetic means and SDs of our flow cytometry data were calculated, using Graph Pad prism 6 (GraphPad, San Diego, CA, USA) software. Data were analyzed using the Student’s *t* test for unpaired samples and error bars in graphs represent the mean ± SEM. Data from bone marrow HSC and VSEL percentages and numbers are expressed as mean ± SD. Differences were analyzed using ANOVA (one-way or multiple comparisons) as appropriate. The significance level throughout the analyses was chosen to be *p* ≤ 0.05.

## Results

### Mouse BM harbors pluripotent VSELs

**Fig. 1:**
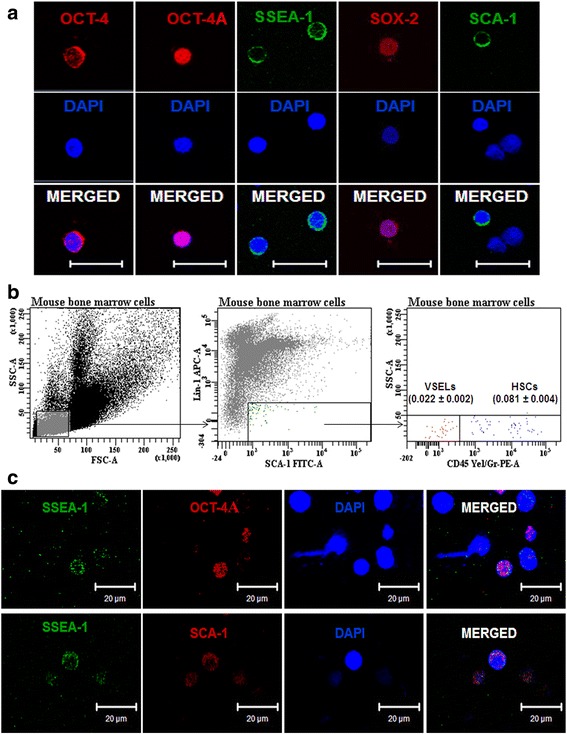
Pluripotent very small embryonic-like stem cells (*VSELs*) in adult mouse bone marrow. **a** Expression of pluripotent stem cell makers including nuclear OCT-4 (*red*) and SOX-2 (*red*) and cell surface SSEA-1 (*green*) and SCA-1 (*green*) were detected on small-sized cells in bone marrow smears. Also note presence of cytoplasmic OCT-4 in slightly bigger cells. These cells were more abundant and please refer to Additional file 1: Figure S1 to see additional images of cytoplasmic OCT-4-expressing cells. Scale bar = 20 μm. **b** Flow cytometry analysis of VSELs and HSCs in bone marrow. Cells of size 2–8 μm were gated using size calibration beads as reference, followed by sequential selection of LIN^–^/SCA-1^+^ cells. This population was evaluated for expression of CD45. The LIN^–^/SCA-1^+^/CD45^–^ cells were VSELs, while LIN^–^/SCA^+^/CD45^+^ cells were HSCs. The average percentage of VSELs and HSCs with SD from 10 experiments is reported. Representative image is shown. **c** Dual immunofluorescence was performed using anti-SSEA1 antibody (*green*) with nuclear OCT-4 (*red*, *upper*) and SCA-1 (*red*, *lower*). Cells co-expressing nuclear OCT-4 and SSEA-1 and SSEA-1 and SCA-1 were observed. Scale bar = 20 μm. In all images, nuclei are counterstained with DAPI. *HSC* Hematopoietic stem cell, *OCT-4* octamer binding transforming factor-4, *SSEA-1* stage-specific embryonic antigen-1, *Sca-1* stem cell antigen-1, *Sox 2* sex-determining region (box 2), *DAPI* 4′,6-diamidino-2-phenylindole (Color figure online)

Earlier reports [6, 53] and our initial immunofluorescence studies on cell smears of mice bone marrow confirmed the presence of rare, small, spherical cells with high nucleo-cytoplasmic ratio expressing pluripotent stem cell markers including nuclear OCT-4A and SOX-2 and cell-surface SCA-1 and SSEA-1 (Fig. 1a). Interestingly, few larger cells with low nucleo-cytoplasmic ratio and prominent cytoplasm were also observed which expressed cytoplasmic OCT-4 (Fig. 1a; Additional file 1: Figure S1). These cells with cytoplasmic OCT-4 were present in more numbers and were possibly the HSCs. Based on these initial results, the BM cells were investigated further by flow cytometry (Fig. 1b) using the protocol and unique gating strategy reported by Kucia et al. [6]. Small cells (2–8 μm) were gated and analyzed for expression of Lineage (LIN) markers and pluripotent stem cell marker (SCA-1). Next, the LIN^–^/SCA-1^+^ cells were gated and analyzed for the expression of hematopoietic marker CD45. The LIN^–^ and CD45^–^ non-hematopoietic cells expressing pluripotent marker SCA-1 were the VSELs (LIN^–^/CD45^–^/SCA-1^+^), while the LIN^–^CD45^+^ hematopoietic cells with SCA-1 expression were the HSCs (LIN^–^/CD45^+^/SCA-1^+^). As shown in Fig. 1b, the percentage of VSELs (0.022 ± 0.002 %) was almost 3-fold lower than the HSCs (0.081 ± 0.004 %) (*n* = 10). A similar strategy was used to determine the percentage of small cells expressing OCT-4A and SSEA-1 and it was observed that the percentage of LIN^–^/CD45^–^ cells co-expressing these markers was almost similar to SCA-1^+^ VSELs (Additional file 1: Table S3). This co-expression of markers in the small cells suggested that SCA-1^+^ VSELs also express OCT-4A and SSEA-1. Dual immunostaining of the bone marrow smears confirmed that cells co-expressed SCA-1 with OCT-4A and SCA-1 with SSEA-1 (Fig. 1c).

From confocal microscopy and flow cytometry studies of intact mouse BM, it was thus confirmed that in addition to the abundant LIN^–^/CD45^–^/SCA-1^+^ HSCs with cytoplasmic OCT-4, mouse bone marrow contained very-small-sized LIN^–^/CD45^–^/SCA-1^+^ VSELs expressing pluripotent markers (nuclear OCT-4 and cell-surface SSEA-1).

### 5-FU treatment spares primitive stem/progenitor cells

**Fig. 2:**
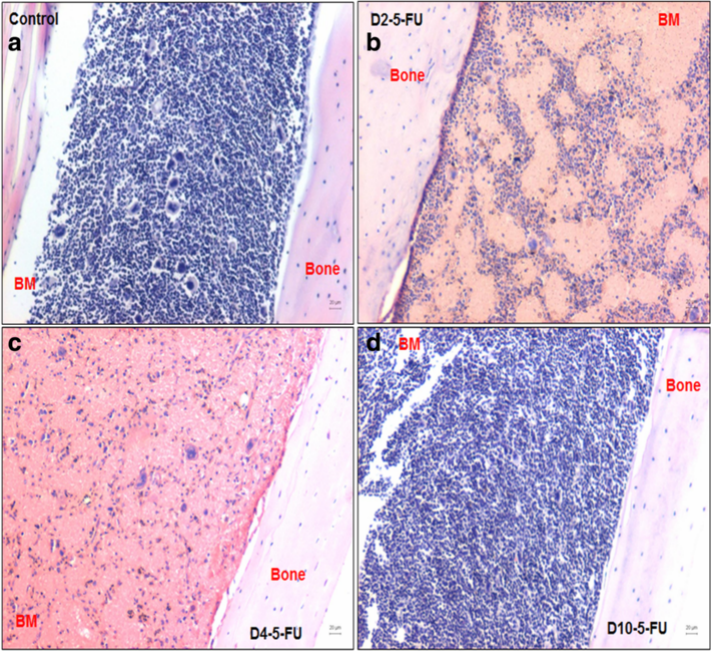
Effect of 5-FU on bone marrow cellularity. Hematoxylin and eosin-stained sections of decalcified femur (**a**) and affected histology on D2, D4, and D10 (**b**–**d**) after 5-FU treatment. Gradual decrease in bone marrow cells is observed on D2 (**b**) and D4 (**c**) with lowest cellularity on D4 (**c**) compared with control (**a**). By D10, endogenous recovery of hematopoiesis is seen (**d**). Note an influx of RBCs on D2 and D4. Scale bar = 20 μm. All images are taken on the Nikon 90i microscope. *D2-5-FU*, *D4-5-FU*, *D10-5-FU* days post 5-fluorouracil treatment

**Fig. 3:**
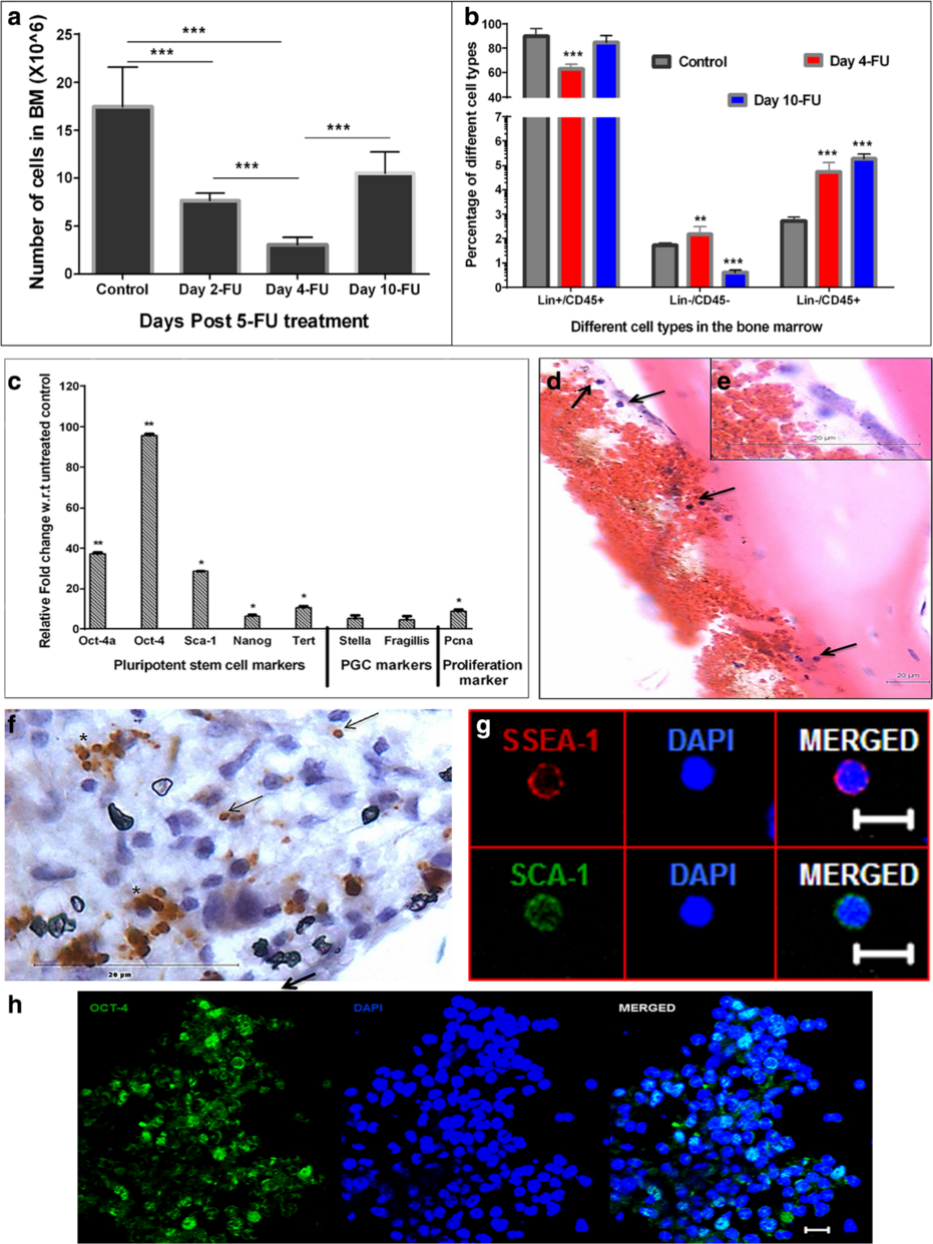
Mature and actively dividing cells in BM are affected by 5-FU whereas primitive and relatively quiescent stem cells are spared. **a** The total BM nucleated cells isolated from two femurs and two tibias of the mice were counted on D2, D4, and D10 after 5-FU treatment and compared with control. Similar to histological data (Fig. 2); the cell count decreased up to D4 (7-fold) and is increased on D10 (*n* = 10, ***p ≤ 0.001). **b** The percentage of various bone marrow subtypes based on Lin and CD45 expression were evaluated on D4 and D10 after 5-FU treatment. Note that while the percentage of Lin^+^/CD45^+^ subtypes decreased on D4, its percentage increased with hematopoietic recovery. Percentage of Lin^–^/CD45^–^ (enriched in VSELs) increased on D4 and slightly reduced by D10. The Lin^–^/CD45^+^ (enriched in HSCs) percentage also increased by D4, and remained high on D10 (*n* = 10, ***p ≤ 0.001). **c** Upregulation in the pluripotent, primordial germ cell and proliferation specific transcripts is observed in BM cells that survive 5-FU on D4. The upregulation of these markers confirms the presence and involvement of primitive stem cells in regeneration. The transcript levels on D4 are shown compared with control (*n* = 6, *p ≤ 0.05; **p ≤ 0.01). Bars in **a**, **b** represent average ± SD and in **c** average ± SEM. **d**–**h** Characterization of cells on D4 that survived 5-FU treatment. **d** H & E staining shows VSELs which are spherical in shape and small in size with high nucleo-cytoplasmic ratio located near the endosteal region. *Inset* (**e**) shows cells at higher magnification. **f** Nuclear OCT-4^+^ VSELs are clearly visualized in bone marrow sections cells in clusters (*) or singly (*arrow*) along the endosteal region; these cells also express (**g**) cell-surface SSEA-1and (**h**) nuclear SCA-1. Presence of nuclear OCT-4, SSEA-1, and SCA-1 confirms that VSELs survive 5-FU treatment. Nuclei are counterstained with DAPI. Scale bar = 20 μm for H & E and immunohistochemistry images, scale bar = 10 μm for immunofluorescence images. *Day 2-FU*, *Day 4-FU*, *Day 10-FU* days post 5-FU treatment, *BM* bone marrow, *5-FU* 5-fluorouracil, *H & E* Hematoxylin and Eosin, *OCT-4* octamer binding transforming factor-4, *SSEA-1* stage-specific embryonic antigen-1, *SCA-1*, stem cell antigen-1, *DAPI* 4′,6-diamidino-2-phenylindole

The effect of 5-FU was studied on mouse BM cellularity by examining the histological sections of decalcified femur post treatment on D2, D4, and D10 along with untreated controls. 5-FU treatment caused an apparent reduction in cell numbers (Fig. 2b) compared with control (Fig. 2a), with the lowest bone marrow cellularity observed on D4 (Fig. 2c); however by D10, BM sections showed similar histoarchitecture to that of the control sections, confirming endogenous regeneration (Fig. 2d). Also, 5-FU treatment resulted in a marked influx of erythrocytes in the bone marrow on D4 (Fig. 2c). Histological observations were further confirmed by BM total cell count taken on the same days. As expected, the cell count steadily decreased by 3-fold on D2 and by 7-fold on D4 (*p* < 0.001) compared with the control (Fig. 3a). The cell count on D4 (3.04 ± 0.79 × 10^6^) was at least 2-fold lower (*p* < 0.001) than on D2 (7.65 ± 0.74 × 10^6^). On D10 post treatment, the cell numbers (10.42 ± 2.3 × 10^6^) were increased, suggestive of endogenous bone marrow regeneration.

We further studied the effect of 5-FU treatment on cell fractions enriched for VSELs (LIN^–^/CD45^–^), HSCs (LIN^–^/CD45^+^), and mature hematopoietic cells comprising myeloid and lymphoid progenitors (LIN^+^/CD45^+^) by flow cytometry. The percentage of these cells was calculated pre and post 5-FU treatment (Fig. 3b). A 1.3-fold decrease in the LIN^+^/CD45^+^ cells was observed compared with control on D4 post treatment (Fig. 3b). However there was an increase in the percentages of both LIN^–^/CD45^–^ (*p* < 0.01) and LIN^–^/CD45^+^ (*p* < 0.001) compared with control on D4 post treatment (Fig. 3b). The results confirmed that 5-FU affects the cycling myeloid and lymphoid compartments while sparing the primitive stem/progenitor populations that are either slow cycling or quiescent. By D10 an increase in the percentage of LIN^+^/CD45^+^ cells was observed with endogenous bone marrow regeneration (Fig. 3b).

Detailed studies were undertaken on the cells that survived in mouse BM on D4 post 5-FU treatment. The quantitative RT-PCR analysis of these cells showed increased expression of pluripotent (Oct-4A, Oct-4, Sca-1 Nanog, and Tert), primordial germ cell (Stella, Fragilis), and proliferation (Pcna) specific transcripts (Fig. 3c). Hematoxylin and eosin (H & E)-stained sections of 5-FU-treated femur on D4 showed the presence of cells with small size, spherical shape, high nucleo-cytoplasmic ratio, and darkly stained nuclei in close proximity of the bone endosteal region (Fig. 3d) These cells expressed nuclear OCT-4 and were observed either singly or in small clusters close to the endosteal region (Fig. 3f). Confocal microscopy studies also demonstrated the expression of SSEA-1, SCA-1 (Fig. 3g), and OCT-4 (Fig. 3h) positive cells in 5-FU-treated smears and cryosections respectively.

From these results it is evident that within 48 h of 5-FU treatment all of the cycling cells in the bone marrow were depleted, and endogenous recolonization of the bone marrow occurred by D10. Characterization studies confirmed that VSELs expressing nuclear OCT4-A and SCA-1 survived the effects of 5-FU treatment.

### VSELs survive and proliferate in response to 5-FU treatment

**Fig. 4:**
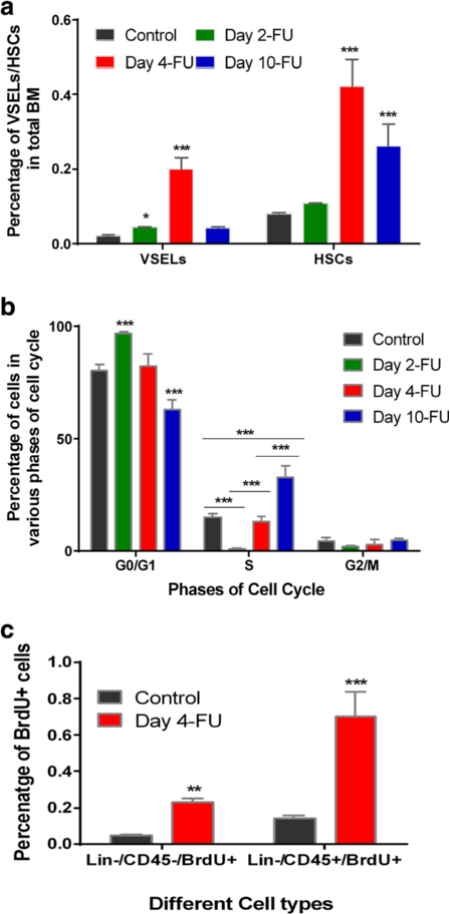
Mouse BM stem/progenitor cells survive and proliferate in response to 5-FU treatment. **a** Flow cytometry analysis of LIN^–^/CD45^–^ VSELs and LIN^–^/CD45^+^ HSCs showed an increase in percentage post treatment compared with control. Compared with D2, a significant increase was observed on D4. On D10 the percentage of both these cells is reduced (*N* = 12, ***p ≤ 0.001). **b** Propidium iodide-based cell cycle analysis of BM cells carried out on D2, D4, and D10 after 5-FU compared with the control showed a decrease in S-phase cells on D2 and an increase in the G_0_/G_1_ cell percentage. By D4 the percentage of S-phase cells increased and continued until D10. The increase in S-phase cells suggests proliferation of cells in response to 5-FU treatment (*N* = 6, ***p ≤ 0.001). **c** Proliferation of VSELs (LIN^–^/CD45^–^) and HSCs (Lin^–^/CD45^+^) was confirmed by an increase in percentage of BrdU-positive cells on D4 compared with control (*N* = 5, **p ≤ 0.01,***p ≤ 0.001). In (**a-c**) Bars represent average ± SD. *Day 2-FU*, *Day 4-FU*, *Day 10-FU* days post 5-fluorouracil treatment, *BM* bone marrow, *VSEL* very small embryonic-like stem cell, *HSC* hematopoietic stem cell, *BrdU* bromodeoxyuridine

Flow cytometry analysis confirmed survival and an increase in the numbers of LIN^–^/CD45^–^/SCA-1^+^ VSELs and LIN^–^/CD45^+^/SCA-1^+^ HSCs after 5-FU treatment. The VSELs were resistant to 5-FU and the percentage of VSELs increased on D2 after treatment (Fig. 4a). However, to confirm that the increase in percentage was not a result of decreased bone marrow cellularity, absolute numbers were calculated (Table 1). These data showed that the VSELs remained constant after treatment whereas more than 50 % of HSCs were destroyed by 5-FU.

**Table 1:** Absolute numbers of VSELs and HSCs in adult mouse bone marrow

Treatment group	VSELs (× 10^3^)	HSCs (× 10^3^)
Control (no treatment)	3.77 ± 1.01	14.07 ± 3.60
Day 2 post 5-FU	3.49 ± 0.45	8.63 ± 0.70
Day 4 post 5-FU	5.89 ± 0.82	12.29 ± 1.59
Day 10 post 5-FU	4.22 ± 0.80	23.73 ± 3.25

*VSEL* very small embryonic-like stem cell, *HSC* hematopoietic stem cell, *5-FU* 5-fluorouracil

The percentage and absolute numbers of VSELs and HSCs were also calculated on D4 and D10 post treatment (Fig. 4a, Table 1). There was an increase in percentage and absolute numbers of VSELs and HSCs on D4 (VSELs 0.201 ± 0.03 %, HSCs 0.423 ± 0.07 %; Table 1). With the increase in BM cellularity by D10, VSELs reduced slightly but HSC numbers were still high (Table 1). Also the percentage of HSCs was always higher compared with VSELs on all of the days. The expansion in VSELs and HSCs on D4 suggested that these quiescent cells were activated and entered the cell cycle in response to the stress induced by 5-FU. An increase in numbers of both VSELs and HSCs was observed with higher HSC numbers compared with the VSELs.

The increase in VSEL and HSC number post treatment (Table 1) suggested that they may be proliferating due to the “regenerative pressure” to meet the increased demand of progenitors. This proliferative status of the bone marrow stem/progenitor cells was studied by evaluating the percentage of cycling cells post 5-FU treatment. The percentage of S-phase cells decreased on D2 of 5-FU treatment, further confirming that 5-FU destroyed cycling cells (Fig. 4b). By D4, the percentage of cells in S-phase increased and was associated with a decrease in the G_0_/G_1_ cell percentage. This increase suggests self-renewal (activation/involvement) of quiescent cells to restore hematopoiesis by D10.

**Fig. 5:**
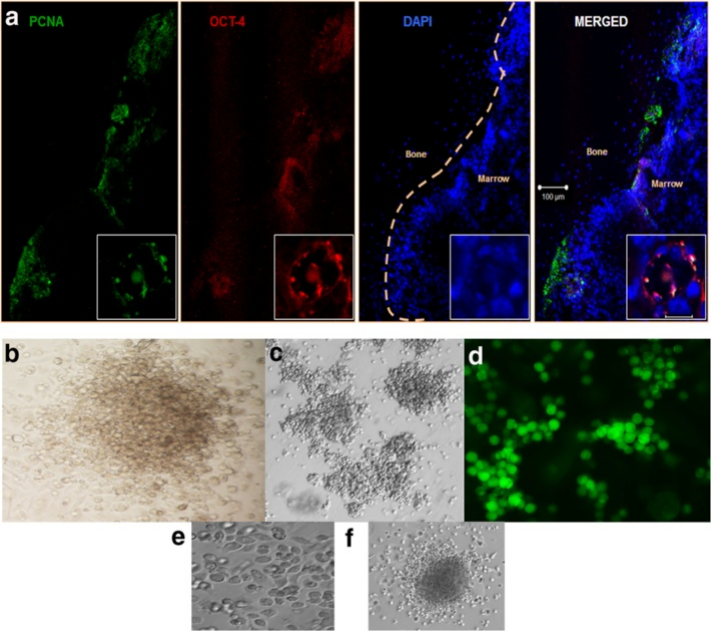
Proliferation of nuclear OCT-4^+^ VSELs. **a** Dual immunofluorescence on cryosections of D4 femur detected the presence of nuclear OCT-4^+^ cells co-expressing proliferation marker PCNA. Nuclear OCT-4/PCNA co-expressing VSELs were observed close to the endosteal region. The nuclei were counterstained with DAPI. Scale bar = 100 μm. *Inset*: magnified image of cluster of OCT-4/PCNA coexpressing cells. Scale bar = 10 μm. **b, c** Results of the CFU assay: small and large hematopoietic colonies of type CFU-GM or CFU-GEMM were observed on culture of cells that survived 5-FU on D4 in Methocult media. **d** GFP^+^ cell clusters observed after 10 days of culture of 5-FU-treated GFP cells in Methocult media. **e** Typical cobblestone formation representative of hematopoietic colonies seen in 5-FU-treated cell culture. **f** Representative image of colonies observed in CFU assay at 20× magnification, colony formed is of the type CFU-GEMM. *OCT-4* octamer binding transforming factor-4, *PCNA* proliferating cell nuclear antigen, *DAPI* 4′,6-diamidino-2-phenylindole, *CFU-GM* colony-forming unit granulocytes/macrophage, *CFU-GEMM* colony-forming unit-granulocyte, erythroid, macrophage, megakaryocyte

An in vivo BrdU incorporation assay was then carried out to examine whether both the LIN^–^/CD45^+^ (enriched in HSCs) and LIN^–^/CD45^–^ (enriched in VSELs) populations proliferated in response to 5-FU treatment. There was a significant increase in the BrdU uptake by both these cell types (*p* < 0.001) on D4 compared with untreated control, confirming their proliferation (Fig. 4c). BM cryosections on D4 showed the presence of VSELs which co-expressed nuclear OCT-4 and PCNA near the endosteal region of the BM (Fig. 5a). Together, the uptake of BrdU by non-hematopoietic cells and the expression proliferation marker PCNA by the pluripotent OCT4-expressing cells confirms that VSELs underwent self-renewal and thus contributed to restoring hematopoiesis by D10. We further examined the functional potential of cells that survived 5-FU treatment in vitro using a colony-forming unit (CFU) assay. Cells that survived 5-FU responded to the cytokines in the Methocult media and developed 20–25 small and large colonies (Fig. 5b–e).

5-FU treatment mirrored a state of stress in the bone marrow with the need to form blood cells to recolonize the marrow. Several groups have already shown that VSELs remain dormant most of the time, entering the cell cycle only under stress and injury [13, 15, 24–30]. Because of 5-FU-induced stress in the present study, VSELs underwent a short burst of proliferation and then by D10 their numbers became equivalent to the control; in contrast, the HSC numbers initially decreased dramatically, but after D4 showed high proliferative potential. This expansion in HSCs with a small but significant increase in VSELs suggests that VSELs may be proliferating to meet increased demand of their immediate progenitor HSCs. These results are in agreement with the stem cell/progenitor concept wherein the most primitive stem cells undergo rare asymmetric divisions to self-renew and give rise to the progenitors which in turn divide rapidly and undergo clonal expansion followed by differentiation to maintain tissue homeostasis.

### FSH enhances hematopoietic recovery from VSELs

**Fig. 6:**
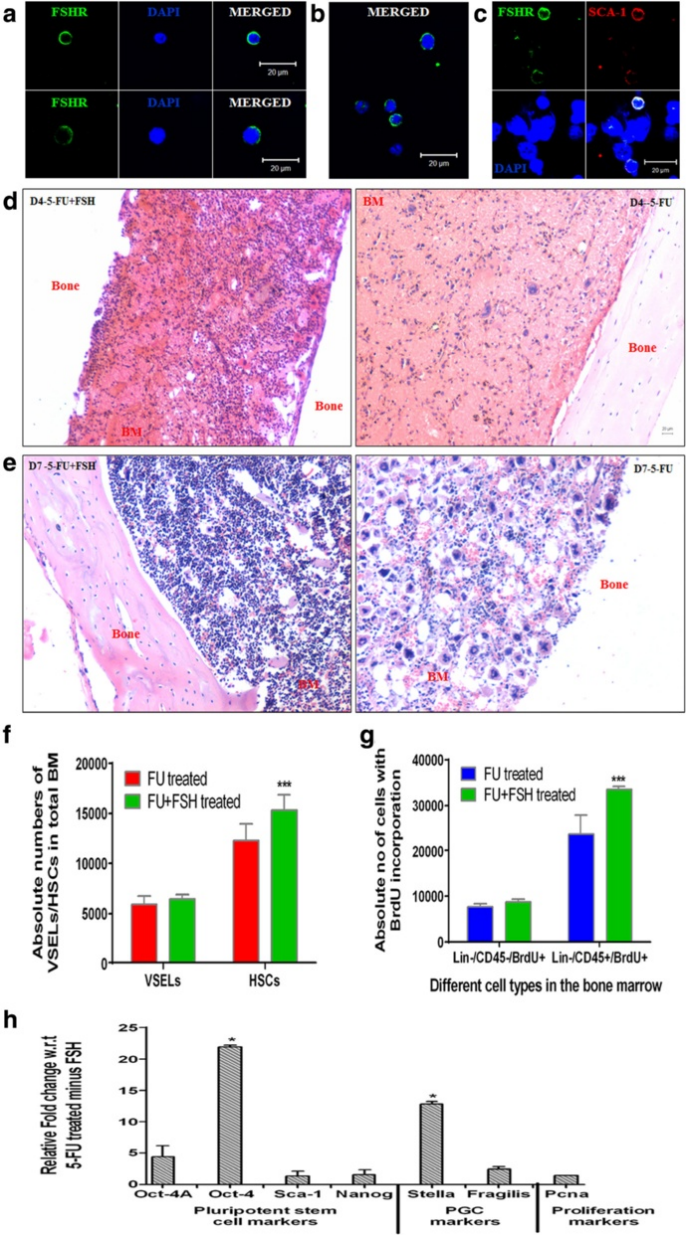
Hematopoiesis recovery is augmented after FSH treatment. **a**, **b** Cell surface expression of FSHR (*green*) on both small VSELs (*top panel*) and slightly bigger HSCs (*bottom panel*) which survive 5-FU treatment in mouse BM on D4. **c** Co-expression of SCA-1 (*red*) and FSHR (*green*) on the same cells. The nuclei are counterstained with DAPI. Scale bar = 20 μm. **d**, **e** H & E sections on D4 showed increased BM cellularity after FSH treatment on D4 (**d**) compared with FSH minus control. **e** In 5-FU + FSH-treated sections, the endogenous bone marrow regeneration was almost complete by D7 after 5-FU treatment compared with untreated controls. **f** Flow cytometry data showed an increase in number of VSELs and HSCs on FSH treatment. **g** BrdU uptake also increased on FSH treatment slightly in the primitive Lin^–^/CD45^–^ (enriched in VSELs) while a significant increase (*p* < 0.001) was seen in Lin^–^/CD45^+^ (HSC-enriched) cells. **h** Upregulation of transcripts specific for pluripotent, primordial germ cells, and proliferation markers on FSH treatment compared with untreated controls. Significant increase in Oct-4 and Stella transcripts was observed. Please note that Oct-4 transcripts (which reflect HSCs) were more than Oct-4A (which reflects VSELs). Bars in **f**, **g** represent average ± SD and in **h** average ± SEM. Representative of five experiments in **f**, **g** and in **h** data obtained from three experiments. *p ≤ 0.05. ***p ≤ 0.001. *D4-5-FU*, *D7-5-FU* days post 5-FU treatment, *FSH* follicle-stimulating hormone, *BM* bone marrow, *5-FU* 5-fluorouracil, *H & E* hematoxylin and eosin, *VSEL* very small embryonic-like stem cell, *FSHR* follicle stimulating hormone receptor, *SCA-1* stem cell antigen-1, *HSC* hematopoietic stem cell, *OCT-4* octamer binding transforming factor-4, *BrdU* bromodeoxyuridine (Color figure online)

To outline the effect of FSH on stem/progenitor cells in the mouse BM, initially the expression of FSHR on the bone marrow cells was studied. Immunofluorescence studies on BM cells that survived 5-FU treatment on D4 showed cell surface expression of FSHR on cells of two distinct sizes (Fig. 6a, b; Additional file 1: Figure S3). Co-expression of SCA-1 and FSHR was also observed, confirming that indeed stem/progenitors express FSHR (Fig. 6c). In addition to FSHR, cells that survived 5-FU treatment also expressed the primordial germ cell (PGC) marker STELLA (Additional file 1: Figure S2) and showed increase in PGC transcripts (Fig. 3c). Immunophenotyping results (Additional file 1: Table S4, Figure S3) showed that only 6 % of LIN^–^/CD45^–^ VSELs and 10 % of LIN^–^/CD45^+^ HSCs expressed FSHR.

We next determined whether the cells surviving in the BM after 5-FU treatment responded to FSH. Recombinant human FSH (5 IU, subcutaneous) was administered 48 h post 5-FU treatment (5-FU + FSH group) and its effects were examined 2 days later (Fig. 6d) and compared with 5-FU-treated mice without FSH (5-FU – FSH group). Analysis of the cell count (data not shown) and H & E stained sections of bone marrow from both the groups showed a slight increase in bone marrow cellularity in response to FSH treatment. Moreover, on D7 post 5-FU treatment the BM architecture of the 5-FU + FSH group showed restoration of bone marrow hematopoiesis (Fig. 6e). This suggested that FSH enhanced the process of hematopoietic recovery by 72 h in the 5-FU + FSH group because complete recovery was noted in the 5-FU – FSH group only by D10.

The stem/progenitor cells in bone marrow (which survived 5-FU) also responded to FSH treatment. There was a small increase in the absolute numbers of VSELs (5890 untreated vs 6455 FSH treated) and a 1.25-fold rise in HSC numbers (*p* < 0.001) after FSH treatment (15,309 FSH treated vs 12,295 untreated) compared with untreated controls (Fig. 6f). To confirm that FSH indeed brought about proliferation of surviving BM stem/progenitor cells and did not just aid in their survival, BrdU incorporation was studied in the LIN^–^/CD45^–^ and LIN^–^/CD45^+^ cells. Similar to our findings in the VSEL and HSC numbers, there was marginal increase (7693 untreated vs 8425 FSH treated) in LIN^–^/CD45^–^/BrdU^+^ cells that are enriched in VSELs along with a remarkable increase (23,699 untreated vs 33,507 FSH treated; 1.41-fold, *p* < 0.01) even in the LIN^–^/CD45^+^ cells (enriched in HSCs, Fig. 6g). The huge expansion in HSCs with minimal increase in VSEL numbers advocates that FSH influences both self-renewal of VSELs and rapid proliferation of progenitors (HSCs) which will then differentiate further to restore hematopoiesis.

The quantitative RT-PCR data of the 5-FU + FSH group showed that treatment also had an effect on the pluripotent stem cell marker expression (Fig. 6h). The transcripts for Oct-4A, Nanog, Sca-1, and Pcna were upregulated compared to the group without FSH, with an almost 21-fold increase (*p* < 0.05) in the expression of Oct-4. FSH treatment also resulted in the upregulation of the transcripts specific for primordial germ cell specific transcripts including a 2-fold increase in Fragilis and a 12-fold increase (*p* < 0.05) in Stella. These results add another level of confirmation to the stimulatory effect of FSH on VSELs and HSCs in the bone marrow post 5-FU.

To conclude, the results of this section show that a fraction of stem/progenitors which survive 5-FU treatment express FSHR and are stimulated by FSH to enhance recolonization by 72 h. A several-fold increase in transcripts specific for Oct-4 (reflecting HSCs) and a marginal increase in Oct-4A (reflecting VSELs) confirmed a larger response of HSCs compared with VSELs which was noted also at the protein level. Further, a 12-fold increase in transcripts specific for Stella vs 2-fold increase in Fragilis suggests that only a fraction of perhaps more committed VSELs express FSHR and respond to FSH. These observations require further investigation.

## Discussion

VSELs were identified and characterized in various adult organs in mice in 2006 [6] and since then several reports have highlighted their presence in human tissues as well, and even their translational potential is beginning to emerge [54–61]. Results of the present study confirm the presence of LIN^–^/CD45^–^/SCA-1^+^ VSELs (0.022 ± 0.002 %) along with LIN^–^/CD45^+^/SCA-1^+^ HSCs (0.081 ± 0.004 %) in mouse bone marrow. VSELs were small in size and co-expressed nuclear OCT-4, SCA-1, and SSEA-1 and pluripotent (Oct-4A, Sca-1, Nanog, Tert) as well as primordial germ cell (Stella, Fragilis) specific transcripts. HSCs were slightly bigger in size, expressed cytoplasmic OCT-4, and were present in relatively greater numbers. VSELs were found to express both pluripotent and primordial germ cell specific markers since it is postulated that they share a developmental link with primordial germ cells [23]. These results are in agreement with our earlier report on human cord blood [11] and with other studies that have reported high expression of primordial germ cell specific transcripts Vasa, Blimp1, Stella, Prdm14, Nanos3, and Fragilis in VSELs [62].

A precaution taken in the present study for detecting VSELs along with the HSCs was the use of a speed of 1000 × *g* to spin-down cells at various steps during processing since VSELs are invariably lost during processing at lower speeds. The inability to detect VSELs in mouse BM in a recent study [63] is most probably because a low speed of 400 xg was used while processing, which probably resulted in loss of VSELs in the supernatant. Similarly VSELs have been invariably and unknowingly discarded along with the RBCs [10] on density gradient centrifugation of cord blood samples. VSELs are endogenous pluripotent stem cells and a possible, novel candidate for regenerative medicine in addition to human embryonic and induced pluripotent stem cells.

One of the main objectives of the present study was to examine the effects of 5-FU on VSELs and to investigate whether VSELs contribute to hematopoiesis in vivo. Under steady-state conditions, VSELs remain quiescent but in response to tissue injury or stress they are activated and mobilize to the affected area [24–30]. A condition of bone marrow stress was thus used in the present study to understand the role of VSELs in bone marrow regeneration. 5-FU, a chemotherapeutic agent, rapidly depletes the cycling myeloid and lymphoid progenitors [64–67]. This creates a dearth of progenitors in the bone marrow and cytotoxic stress is established. Additionally, treatment with 5-FU spares the slow cycling and dormant cells, thereby resulting in bone marrow enriched in primitive stem cells [68–71]. Even in the present study, as expected 5-FU caused 7-fold depletion in bone marrow cellularity by D4 and spontaneous endogenous recovery of the bone marrow occurred by D10, which established a basal timeline for the later experiments.

The kinetics of VSELs and HSCs was studied further within this period of 2–10 days post treatment. VSELs, being the most primitive cells in the bone marrow and quiescent under normal conditions, were resistant to the effects of 5-FU confirmed by no change in their numbers on D2 (3.49 ± 0.45 × 10^3^) compared with untreated control (3.77 ± 1.01 × 10^3^). On the other hand, more than 50 % of HSCs were depleted in response to 5-FU (8.63 ± 0.70 × 10^3^) on D2 compared with untreated control (14.07 ± 3.60 × 10^3^) in agreement with earlier reports [67, 69, 72]. The bias in the effect of 5-FU on HSCs was possibly due to the presence of two separate sub-populations within the HSCs, comprising of the quiescent cells that survive 5-FU cytotoxicity and the actively dividing cells which get depleted. These two sub-populations have been described as long- and short-term repopulating subsets of HSCs which show varying degree of radioprotection [73].

The increase in numbers of both VSELs and HSCs in response to 5-FU confirmed at both protein and mRNA levels and by BrdU and cell cycle analysis in the present study suggests that the stem/progenitor cells get activated in response to stress, and both proliferate but the extent of proliferation of HSCs is much higher compared with VSELs. This compensatory expansion of stem/progenitors initiates regeneration of chemoablated BM. Our results are in agreement with earlier reports by Ratajczak et al. [31], who reported BrdU incorporation in 12 % of the VSELs post irradiation in mouse bone marrow. Similarly, Baldrige et al. [74] also reported activation of both intermediate blood progenitors and primitive long term-HSCs (LT-HSCs) after chronic bacterial infection. Conclusive evidence that VSELs survive and proliferate in BM after 5-FU treatment is provided by us for the first time in the literature by dual immunofluorescence studies on BM sections. Nuclear OCT-4A and PCNA-expressing VSELs are clearly evident on D4 along the endosteal region of the BM which is considered to be the niche for stem cells [75, 76]. The niche maintains stem cells pool in vivo and regulates the hematopoietic recovery after myelo-suppression [77–79]. The regenerative ability of cells that survive chemoablation in BM was also confirmed by CFU assay in vitro that resulted in colony formation in Methocult media, thus implying that surviving 5-FU cells are functional and can form mature blood lineages to recolonize the BM.

Furthermore, we report two distinct sizes of cells (possibly VSELs and HSCs) expressing FSHR in ablated BM. Similar expression of FSHR on stem/progenitors has been reported in mouse BM and cord blood [46, 47] and on ovarian and testicular stem/progenitors cells by our group [49–52]. However further studies are required to examine this in a more quantitative manner because we found only 6 % of LIN^–^/CD45^–^ VSELs and 10 % of LIN^–^/CD45^+^ HSCs expressing FSHR by immunophenotyping studies. Our group has earlier shown that FSH treatment stimulates ovarian and testicular VSELs to self-renew in chemoablated gonads and also stimulates clonal expansion of progenitors [15, 50, 52]. Recently, Zbucka-Kretowska et al. [48] reported that FSH mobilized BM stem/progenitor cells into circulation but not endothelial progenitor cells in 15 female patients on FSH therapy for ovarian stimulation in an infertility clinic. The augmented hematopoietic recovery on D7 in response to FSH treatment noted in the present study after compared with D10 in untreated mice provides further evidence that indeed FSH influences stem/progenitor cells in BM. The enhanced recovery on FSH treatment was found to be accompanied with a marginal increase in the number of VSELs and a dramatic increase in HSCs and differentiated cells.

True stem cells are expected to divide asymmetrically (ACD) and give rise to two distinct cell types, whereas the progenitors that arise by ACD of stem cells undergo rapid symmetric divisions (SCD) and differentiation to maintain tissue homeostasis. Although the HSCs are the best investigated human somatic stem cells, which cells undergo ACD is still not clearly understood [80]. Emerging evidence suggests that (1) VSELs are the most primitive small-sized stem cells in the BM in addition to actively dividing, larger sized HSCs, (2) VSELs give rise to HSCs based on in-vitro studies [31, 32] and the OCT-4 expression pattern shown in the present study wherein VSELs express nuclear OCT-4A and HSCs express cytoplasmic OCT-4, and (3) both VSELs and HSCs are stimulated and take part in regeneration of BM under stress conditions shown in the present study and also reported earlier [13, 15, 31]. Based on this we suggest that VSELs may correspond to the LT-HSCs. A recent report of a similar imprinting pattern of erasure at the IgF2-H19 locus to maintain quiescence in LT-HSCs [81] and VSELs [82] provides further evidence of a link between these cells. VSELs are probably the true stem cells in the hematopoietic system that undergo ACD to self-renew and give rise to HSCs which are actively dividing progenitors that further differentiate into various blood cell types. This was recently discussed in detail in various adult organs [83].

Certain changes in the niche may push the stem cells to undergo uncontrolled proliferation resulting in cancer/leukemia. If this was true the stem cells and cancer cells should share similar markers. Recently Zhao et al. [84] found a significantly increased nuclear OCT-4A expression in patients with acute leukemia. This observation combined with current understanding that VSELs are the most primitive cells expressing nuclear OCT-4A, it can be hypothesized that in the case of leukemia, changes in the niche push the relatively quiescent VSELs to undergo uncontrolled proliferation. This link between VSELs and HSCs and leukemia stem cells warrants further investigation.

## Conclusions

To summarize, bone marrow houses pluripotent VSELs which give rise to the HSCs. VSELs and a subset of HSCs survive 5-FU-induced stress and are activated to recolonize the chemoablated BM. VSELs/HSCs also express FSHR and thus FSH treatment expedites BM regeneration by almost 72 h. This opens up a newer avenue for use of FSH in the clinical setting while treating bone marrow failure and irradiation patients. Our results provide better understanding towards the stem/progenitor cell hierarchy in the hematopoietic system. Studying the factors released by the niche cells which activate stem/progenitor cells to regenerate BM cells under stress conditions and also which result in uncontrolled proliferation and expansion of Oct-4A^+^ VSELs in leukemia patients is warranted.

## Additional file

Additional file 1: Description of methods, primers and antibodies used in the study. (PDF 340 kb)
