# Supplementary material for: Delineating the effects of 5-fluorouracil and follicle-stimulating hormone on mouse bone marrow stem/progenitor cells
Source: Stem Cell Res Ther. 2016 Apr 19;7:59. doi: 10.1186/s13287-016-0311-6 (PMC4837595; doi:10.1186/s13287-016-0311-6)
Supplement: Additional file 1: — Description of methods, primers and antibodies used in the study. (PDF 340 kb) [file 13287_2016_311_MOESM1_ESM.pdf]

Additional Table S1: Details of antibodies used in the present study

Antibody	Description	Company
**LIN-1-APC**<br>CD3e, clone 145-2C11<br>CD11b, clone M1/70<br>CD45R/B220, clone RA3-6B2<br>Ly-76, clone TER-119<br>Ly-6G and Ly-6C clone RB6-8C5	The APC Mouse Lineage Antibody Cocktail has been designed to react with cells from the major hematopoietic lineages, such as T lymphocytes, B lymphocytes, monocytes/macrophages, NK cells, erythrocytes, and granulocytes. This cocktail of five APC-conjugated antibodies is designed for identification of hematopoietic progenitors in mouse bone marrow.	BD Bioscience, San Jose, CA, USA<br>Catalogue no : 558074
**CD45-PE**<br>Clone:30-F11	CD45 is a transmembrane glycoprotein which is expressed at high levels on the cell-surface, and its presence distinguishes leukocytes from non-hematopoietic cells.reported to react with all isoforms and both alloantigens of CD45, which is found on hematopoietic stem cells and all cells of hematopoietic origin, except erythrocytes.	BD Bioscience, San Jose, CA, USA<br>Catalogue no : 553081
**SCA-1-FITC**<br>**SCA-1(*)**<br>Clone E13-161.7	Reacts with Ly-6A.2 and Ly-6E.1, which are allelic members of the Ly-6 multigene family. Sca1 (Ly-6A/E), a phosphatidylinositol-anchored protein of expressed on the multipotent hematopoietic stem cell (HSC) in mice.Sca-1+ HSC are found in the adult bone marrow and fetal liver.	BD Bioscience, San Jose, CA, USA<br>Catalogue no : 553335 (*)<br>Biolegend, San Diego, CA,USA Catalogue no : 122501
**OCT-4**	Transcription factor that Forms a trimeric complex with SOX2 on DNA and controls the expression of a number of genes involved in embryonic development. Critical for early embryogenesis and for embryonic stem cell pluripotency.	Abcam, Cambridge,UK<br>ab19857
**SSEA1**<br>Clone MC-480	Stage-specific embryonic antigen-1 (SSEA-1) is expressed on the surface of early mouse embryos, murine embryonal carcinoma cells (EC), murine embryonic stem cells (ES) and murine & human germ cells (EG).	Millipore, CA,USA<br>Catalogue no : MAB4301
**SOX-2**	SOX-2, transcription factor that forms a part of transcriptional network with Nanog and Oct-4 to maintain cells in a pluripotent state	Cell Signaling, MA, USA<br>Catalogue no : D6D9 (mAb #3579)
**STELLA**<br>(Clone:3H5.2)	Stella is maternal effect gene required for normal early development. It is expressed in primordial germ cells, oocytes, preimplantation embryos, and pluripotent stem cells.	Millipore, CA,USA<br>Catalogue no : MAB4388
**PCNA**<br>Clone PC 10	Found in the nucleus and is a cofactor of DNA polymerase delta. The encoded protein acts as a homotrimer and helps increase the processivity of leading strand synthesis during DNA replication. Marks proliferating cells	Sigma- Adrich,MO,USA<br>Catalogue no : P8825
**FSHR**	Receptor for follicle-stimulating hormone. The activity of this receptor is mediated by G proteins which activate adenylate cyclase.	Abcam, Cambridge,UK<br>ab150557

Additional Table S2: Primers used in the study for qRT-PCR studies

Primer	Sequence
**Oct-4A (F)**	AACCGTCCCTAGGTGAGCCG
**Oct-4A (R)**	CCCACCTGGAGGCCCTTGGAA
**Oct-4 (F)**	CCTGGGCGTTCTCTTTGGAAAGGTG
**Oct-4 (R)**	GCCTGCACCAGGGTCTCCGA
**Nanog (F)**	CAGGAGTTTGAGGGTAGCTC
**Nanog (R)**	CGGTTCATCATGGTACAGTC
**Sca-1 (F)**	AGAGGAAGTTTTATCTGTGCAGCCC
**Sca-1 (R)**	TCCACAATAACTGCTGCCTCCTGA
**Tert (F)**	GAGGTGCAGCGGGATGGGTTG
**Tert (R)**	CACCCAGGGTACCAGGCTCCA
**Stella (F)**	ACGCTTTGGATGATACAGACGTCC
**Stella (R)**	GCGCTTTGAACTTCCCTCCGGA
**Fragilis (F)**	GGGGTGACTGAGCTGGGGGAA
**Fragilis (R)**	TGTCCCTAGACTTCACAGAGTAGGC
**Pcna (F)**	GATGCCGTCGGGTGAATTTG
**Pcna (R)**	TCTCTATGGTTACCGCCTCCT
**18s (F)**	GGAGAGGGAGCCTGAGAAAC
**18s (R)**	CCTCCAATGGATCCTCGTTA

Additional Materials and Methods

Cell cycle analysis: For cell cycle analysis, cells from normal and 5-FU treated mice obtained on D4 and D10 were suspended in 0.5ml of cold hypotonic solution containing 0.1%sodium citrate and 0.3µl/ml of NP-40 (Sigma), 2mg/ml RNase A (Banglore-Genei, India) and 50µg/ml PI (Sigma-Aldrich). The tubes were placed in dark at 4˚C. Analysis by flow cytometry was performed after a minimum of 20 min incubation, but within an hour of hypotonic treatment. Fluorescent events were acquired on a BD FACS Aria flow cytometer and analysed using with BD FACS DIVA software.

Colony forming unit assay: For this BM cells which survived were isolated on D4 after 5-FU treatment to GFP mice. They mostly comprised dividing VSELs (and few HSCs) as shown by BrdU uptake (Figure 4C). These cells were suspended in semisolid methyl cellulose (MethoCult) based medium (GFM3434Stem Cell Technologies,British Columbia, Canada) containing cytokines like SCF, IL6, IL3 and erythropoietin. $1x10^4$ cells were suspended per well in a 6 well dish. Cultures were maintained at 37 C for 2 weeks. Colony forming units were visualized and counted

Bone marrow cell smears: Cell smears of normal and 5-FU treated BM cells were prepared on poly-l-lysine coated glass slides. The smears were air dried and fixed with 4% paraformaldehyde (PFA, Sigma-Aldrich) and slides stored at 4C until use.

Histology Femur bones of normal, 5-FU and 5-FU+FSHtreated mice were collected, decalcified (96 hours) and fixed in 10% formalin + PFA solution for histology and immunolocalization studies. Fixed samples were processed and embedded in paraffin using standard protocols and 5 µm paraffin sections were prepared. Histology of bone sections was studied by staining with Hematoxylin and Eosin (H&E). The representative areas were photographed using Nikon 90i microscope (Nikon, Japan).

Immunophenotyping

The bone marrow cells were incubated in FCR block (20 min at 4°C,Stem cell technologies, Canada) to block nonspecific Fc receptors. Direct immunolabeling was performed with FITC, conjugated SCA-1 antibody (BD Biosciences; 20 min at RT) while in-direct labeling was performed with primary antibodies: anti-OCT-4 (Abcam, Cambridge, UK), anti-SSEA-1(Millipore) , anti-SOX-2 (Cell Signaling) and anti-FSHR (Abcam; 20 min at RT). For cell membrane permeabilization in case of OCT-4 and SOX-2 cytofix/cytoperm buffer (BD

Biosciences) was used. Secondary antibody labeling (20 min at RT) was done using Alexafluor goat anti-mouse/rabbit IgG 568 or IgG 488 (Molecular Probes, Invitrogen). Following appropriate washing procedures, fluorescent events were acquired on BD FACSAria flow cytometer and analysed using with BD FACS DIVA software.

Immunolocalization Studies

*Immuno-histochemistry***:** Sections were deparaffinized and later dehydrated in graded methanol series, endogenous peroxide was blocked using 3% H_2O_2, and antigen retrieval was performed in citrate buffer with a pH of 6 using a microwave. These steps were followed by blocking, primary antibody incubation, and detection using appropriate Vecta ABC elite detection kits (Vector Laboratories Inc,USA). The primary antibody used was anti-OCT4 (Abcam), antibody. The protocol for negative control was exactly identical, except that primary antibody was replaced by blocking solution. The representative areas were photographed using Nikon 90i microscope (Nikon, Tokyo, Japan).

*Immuno-fluorescence***:** Fixed cell smears and cryosections of normal and 5-FU treated bone marrow cells were used. For cryosections, femurs were fixed in Tissue-Tek OCT compound (MILES, USA) and frozen at -80C. 5 µm thick cryosections were cut on Leica CE microtome (Germany). Further, these frozen sections were fixed with 4% PFA for 1 hour, washed with chilled acetone followed by PBS. The smears and cryosections were then washed with wash buffer (PBS containing 0.3% bovine serum albumin and 0.1mM EDTA), permeabilized by 0.3% Triton X-100 for 10 min, again washed in PBS, pre-blocked with 1%BSA and 5% normal goat serum (Bangalore Genei, Bangalore, India) and subsequently incubated with antibodies. Various antibodies used in the study included anti-OCT-4 (Abcam), anti-SSEA-1 (Millipore, USA), anti-SCA-1 (FITC tagged; BD, Purified SCA-1, Biolegend, USA), anti-PCNA (Sigma-Aldrich) and anti-STELLA (Millipore). Permeabilization with Triton X-100 was omitted for cell surface staining (SSEA-1 and SCA-1). The blocking for mouse monoclonal antibodies (STELLA, PCNA) for both single and dual staining was done using M.O.M. detection Kit according to the manufacturer’s instructions (steps 7-9 in their protocol). For detection of OCT-4, STELLA, SSEA-1,SCA-1 and PCNA, cell smears were incubated with secondary antibody (Alexafluor goat anti mouse IgG 568; Alexafluor donkey anti rabbit 568, Molecular probes, USA) in 1:500 dilution however this step was omitted while using FITC tagged SCA-1. Negative controls were incubated in the blocking solution with the omission of primary antibody. Counterstaining was

done using 4, 6-diamidino-2-phenylindole (1mg/mL; DAPI, Molecular Probes) or propidium iodide (PI, Sigma-Aldrich). All images were captured by laser scanning confocal microscope (Carl Zeiss, Germany) using 63X objective.

Quantitative Polymerase Chain Reaction (qRT-PCR) studies

RNA extraction and cDNA synthesis: Total RNA was isolated from BM cells of normal, 5-FU treated and 5-FU+FSH mice using Qiagen RNA extraction Kit (Qiagen Inc, USA), according to the manufacturer's instructions. The extracted RNA was treated with ribonuclease-free deoxyribonuclease (DNase 1; Qiagen). 1µg of total RNA was reverse transcribed using the iScript RT Kit (Bio-Rad Laboratories,USA) according to the manufacturer's instructions to synthesize the first-strand cDNA in a GSTORM thermo cycler (Gene Technologies, Braintree, UK).

qRT-PCR: This was done using iQSYBR Green SuperMix (Bio-Rad). The primers used and their annealing temperatures are mentioned in additional Table 2. All the primers had efficiency close to 100%. Expression of various pluripotent markers viz. Oct-4A, Oct-4, Nanog, Sca-1, Tert; primordial germ cell markers Stella, Fragilis; and proliferation marker Pcna was studied by Q-PCR using a CFX96 real-time PCR system (Bio-Rad Laboratories) and SYBR Green chemistry (Bio-Rad Laboratories). The amplification conditions were as follows: initial denaturation at 95˚C for 5 min, followed by 40 cycles comprising denaturation at 95˚C for 10 s, primer annealing at 62˚C for 30 s, and extension at 72˚C for 30 s. The threshold cycle (Ct) was determined subsequently using the CFX Manager software (Bio-Rad Laboratories) and normalized to the housekeeping gene (18S RNA). Relative fold change of mRNA over the calibrator was expressed as 2^-DDCt, where DCt = Ct of target genes (e.g.OCT4, NANOG) - Ct of endogenous control gene (18s), and DDCt = Normalized Ct of samples for target gene – Normalized Ct of calibrator. Melt curve analysis was performed at the end of every run to confirm the homogeneity of the PCR products, which was also confirmed by running the same on 2% agarose gel (Bangalore Genei).

Absolute number calculation: The absolute number of cells in each population was calculatedbased on the total number of cells recovered from twofemurs and tibias per mouse and the frequency of the cells as determined by flow cytometry

Additional Figures and Tables

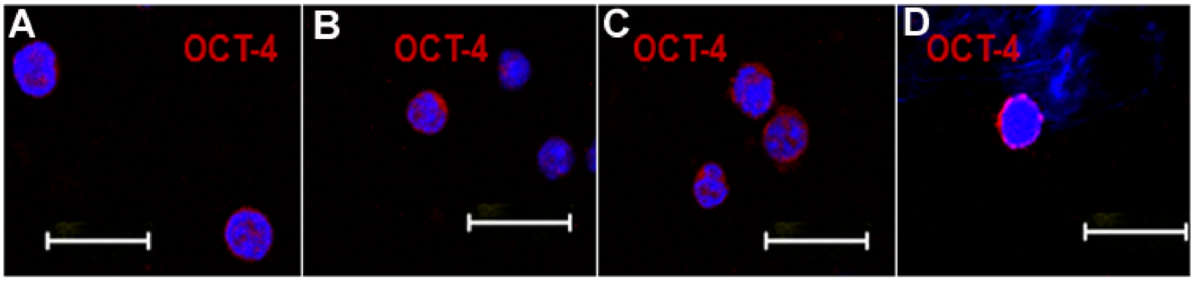

Additional Figure S 1: Evaluation of expression of OCT-4 in the bone marrow. Cells expressing cytoplasmic OCT-4 were slightly bigger compared to small sized VSELs with nuclear OCT-4. The number of cells expressing cytoplasmic OCT4 was greater compared to VSELs expressing nuclear OCT-4, suggesting more number of progenitor population. Scale Bar = 20µm.

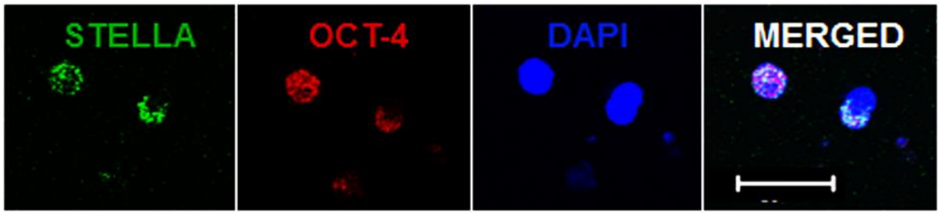

Additional Figure S 2: Bone marrow has cells expressing PGC marker STELLA. Dual immunostaining shows presence of cells co-expressing nuclear OCT-4 (red) and PGC marker STELLA (green). Nucleus counterstained with DAPI. The co-expression of these markers shows a developmental link between VSELs and primordial germ cells, as reported earlier by other group. Scale bar = 20µm.

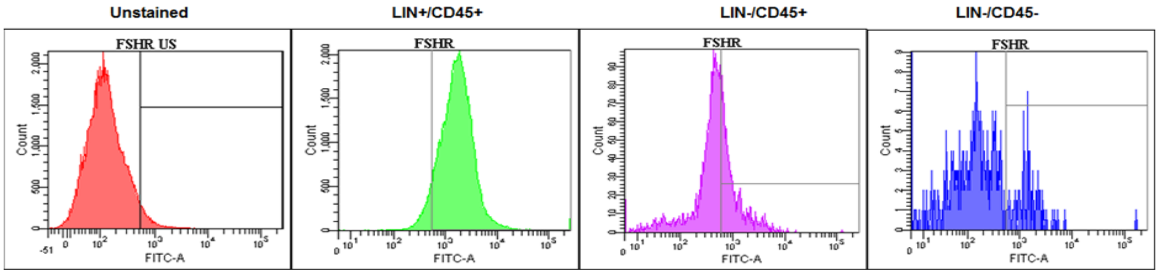

Additional Figure S3: Flow cytometry based evaluation of FSHR on bone marrow cells. All the sub types (LIN+/CD45+; LIN-/CD45+ and LIN-/CD45-) expressed FSHR. The percentage of FSHR+ cells were determined by the shift in FITC peak as compared to unstained. Lowest expression was seen among the LIN-/CD45- cells. Data analyzed using FACS Diva software. Representative image of three experiments.

Additional Table S3

Percentage of LIN-/CD45- cells expressing pluripotent stem cell markers

Pluripotent Stem Cell Marker	Percentage of Lin-/CD45- cells expressing pluripotent stem cell markers
**OCT -4**	0.020 ± 0.002
**SSEA-1**	0.022 ± 0.003
**SCA-1**	0.022 ± 0.002

OCT-4, Octamer binding transforming factor-4; SSEA-1, Stage specific embryonic antigen-1; Sca-1, Stem cell antigen-1

Additional Table S4

Expression of FSHR on various cell types in the mouse bone marrow

Cell type	Percentage of FSHR positive cells
LIN+/CD45+ (Myeloid and lymphoid Cells)	61.34 ± 1.65
LIN-/CD45+ (HSCs)	10.17 ± 1.22
LIN-/CD45- (VSELs)	6.193 ± 2.88

FSHR, Follicle Stimulating Hormone receptor; VSELs, very small embryonic-like stem cells; HSC, Hematopoietic Stem Cells
